# Low prevalence of myocilin mutations in an African American population with primary open-angle glaucoma

**Published:** 2012-08-10

**Authors:** Wenjing Liu, Yutao Liu, Pratap Challa, Leon W. Herndon, Janey L. Wiggs, Christopher A. Girkin, R. Rand Allingham, Michael A. Hauser

**Affiliations:** 1 Center for Human Genetics, Duke University Medical Center, Durham, NC; 2 Department of Ophthalmology, Duke University Eye Center, Durham, NC; 3 Department of Ophthalmology, Harvard Medical School and Massachusetts Eye and Ear Infirmary, Boston, MA; 4 Department of Ophthalmology, University of Alabama at Birmingham, Birmingham, AL

## Abstract

**Purpose:**

Mutations in the myocilin gene (*MYOC*) are associated with primary open-angle glaucoma (POAG) in many different populations. This study represents the first large survey of *MYOC* mutations in an African American population.

**Methods:**

We recruited 529 African American subjects with POAG and 270 African American control subjects in this study. A complete eye examination and blood collection was performed in all study subjects. Genomic DNA was extracted. The entire coding sequence of *MYOC* was amplified and sequenced using the Sanger method. Identified *MYOC* variants were compared with previously reported *MYOC* mutations.

**Results:**

We identified a total of 29 *MYOC* variants including six potential *MYOC* mutations. Two mutations (Thr209Asn and Leu215Gln) are novel and are found only in cases and no controls. We also identified four previously reported *MYOC* mutations in cases and no controls (Tyr453MetfsX11, Gln368X, Thr377Met, and Ser393Arg). The overall frequency of glaucoma-causing *MYOC* mutations in our African American population with POAG was 1.4%.

**Conclusions:**

We identified two novel probable glaucoma-causing *MYOC* mutations (Thr209Asn and Leu215Gln). This study indicates that, despite the high prevalence of POAG, *MYOC* mutations are rare in the African American population.

## Introduction

Glaucoma is the leading cause of irreversible blindness in the world and the second most common cause of permanent blindness in the USA [1-3]. Glaucoma is a heterogeneous group of disorders characterized by optic nerve damage, progressive loss of retinal ganglion cells, and specific visual field defects [4,5]. Of the many types of glaucoma, primary open-angle glaucoma (POAG) is the most common [5]. The prevalence of POAG differs among populations. African-Americans are four to five times more likely to be affected by POAG than Caucasian Americans [6].

Genetic defects have been shown to contribute to the pathogenesis of POAG through family linkage analysis and case-control based association studies [5,7-9]. At least 14 chromosomal loci for POAG have been reported [4,5]. To date, several causative genes for POAG from these 14 POAG-associated loci have been identified, including myocilin (*MYOC*) [10,11], optineurin (*OPTN*) [12], WD repeat domain 36 (*WDR36*) [13], cytochrome P450 1B1 (*CYP1B1*) [14], and TANK-binding kinase 1 (*TBK1*) [15,16] (Human Genome Organization). Among these genes, *MYOC* has been found to harbor the greatest number of glaucoma-causing mutations with over 80 mutations identified in different populations [17,18].

*MYOC* is composed of 3 exons. Its protein product MYOC is broadly expressed [18-20]. The first exon encodes a peptide sequence similar to muscle protein myosin and the third exon encodes a peptide sequence homologous to olfactomedin [21]. The majority of myocilin mutations are missense variants located in the third exon that are thought to affect the solubility and disrupt the secretion of the protein [22,23]. Despite extensive research, it remains unclear how myocilin mutations lead to glaucoma [4,8,20,24,25].

Myocilin mutations have been found with an overall frequency of 2%–4% in all populations worldwide [5,8,18,24,26]. In African populations, probable disease-causing myocilin mutations were found in 1.75% of Moroccan POAG subjects and 4.4% of Ghanaian and South African POAG subjects [27-29]. Caucasian populations have been studied extensively and the frequency of myocilin mutations in adult-onset POAG subjects has been found to be 2 to 5% [30-35]. Thus far, only one study has looked at the frequency of myocilin mutations in an African American population and found 2.6% of 312 African American POAG patients harbored probable disease-causing mutations [30]. Our study represents the largest survey of African American POAG and control subjects for myocilin mutations in all 3 exons of the myocilin gene.

## Methods

### Study subjects

The study adhered to the tenets of the Declaration of Helsinki. Informed consent was obtained from all study participants. The research was reviewed and approved by the Institutional Review Board from all participating institutions, including both Duke University Medical Center (Durham, NC) and the Massachusetts Eye and Ear Infirmary (Boston, MA). Study subjects were recruited from the Duke University Eye Center (Durham, NC) and the Massachusetts Eye and Ear Infirmary (Boston, MA) for a total of 529 African American subjects with POAG and 270 African American control subjects. Subjects with POAG were unrelated and met the following inclusion criteria: 1) age of onset greater than 18 years; 2) glaucomatous optic neuropathy in both eyes; and 3) visual field loss consistent with optic nerve damage in at least one eye. Glaucomatous optic neuropathy was defined as a cup-to-disc ratio greater than 0.7 or focal loss of the nerve fiber layer resulting in a notch, associated with a glaucomatous visual field defect. Visual fields were performed using standard automated perimetry or frequency doubling test. An open anterior chamber angle has been found in all the POAG cases. IOP was not used as an inclusion criterion. The exclusion criteria for POAG subjects included the diagnosis of a secondary form of glaucoma or a history of ocular trauma. The control subjects were examined by a board-certified ophthalmologist. The control subjects were unrelated and met the following criteria: 1) no first-degree relative with glaucoma; 2) IOP less than 21 mmHg in both eyes without treatment; 3) no evidence of glaucomatous optic neuropathy in either eye; and 4) normal visual field in both eyes. The normal controls were recruited specifically for this study and visual field was included in the eye exam.

### Genomic DNA sequencing

Genomic DNA was extracted using standard methodology as previously described [36-38]. Briefly, genomic DNA was purified using a modified salting-out method. Primers flanking the entire coding sequence of *MYOC* were designed with Primer3 software [39]. Primer sequences are provided in Table 1. The amplified region covered at least 50 base pairs into each intron to screen for potential mutations affecting exon splicing. Platinum Taq DNA polymerase (Invitrogen, Carlsbad, CA) was used for all of the polymerase-chain reactions (PCR). The PCR amplifications were performed in TherymoHybaid MBS PCR machines (Thermo Scientific, Waltham, MA). Completed PCR reactions were purified and sequenced in the forward direction using BigDye chemistry (Applied Biosystems, Carlsbad, CA). Potential mutations were confirmed by additional sequencing in the reverse direction. All the sequences were analyzed using the Sequencher 4.9 software package (Gene Codes, Ann Arbor, MI). The Fisher’s exact test was used to test the association of all variants with POAG.

**Table 1:** List of PCR primers for *MYOC* (myocilin) exon sequencing in African American POAG subjects and controls.

***MYOC* exon**	**Forward primer sequence**	**Reverse primer sequence**	**Amplicon size (bp)**	Covered Genomic Region*
Exon1a	ATCTTGCTGGCAGCGTGAA	TCTCTGGTTTGGGTTTCC	614	chr1:171,621,342–171,621,955
Exon1b	GACAGCTCAGCTCAGGAAGG	GAAGGTGATCGCTGTGCTTT	663	chr1:171,620,991–171,621,653
Exon2	AGCAAAGACAGGGTTTCACC	AGGGCTTTGTTAGGGAAAGG	554	chr1:171,607,517–171,608,071
Exon3a	CCCAGACGATTTGTCTCCAG	TCCCAGGTTTGTTCGAGTTC	648	chr1:171,605,327–171,605,974
Exon3b	GAGAAGGAAATCCCTGGAGC	TGGTGACCATGTTCATCCTTC	598	chr1:171,604,914–171,605,511

* The covered genomic regions were based on the February 2009 human reference sequence (GRCh37). POAG is for primary open-angle glaucoma. bp is for base pair.

## Results

The study data set was composed of 529 African American subjects with POAG and 270 African American control subjects. The mean age of diagnosis in the subjects with POAG was 54.8 years and 42.5% of these subjects were female. In comparison, the mean age of the control subjects was 57.5 years (ranging from 40 to 85 years) and 53.3% of the control subjects were female.

We identified a total of 29 sequence variants, including 28 coding variants. Of these 29 variants, we identified four known myocilin mutations (Table 2), including two nonsense mutations (Tyr453MetfsX11 and Gln368X) and two missense mutations (Thr377Met and Ser393Arg). These 4 mutations are all located in exon 3. Each mutation was found in 1 POAG patient and no controls. They have all been previously reported as glaucoma-causing mutations [17]. We also identified two novel missense mutations: Thr209Asn in one POAG patient and Leu215Gln in two POAG patients, but not in controls (Table 2). Both of these novel mutations are located in exon 2. Combining all the patients with myocilin mutations, we have identified six different myocilin mutations in 7 African American POAG patients and no controls, accounting for approximately 1.4% of all the POAG patients. These African American patients carrying myocilin mutations tend to have advanced glaucoma with high IOP and large cup-to-disc ratio (Table 2).

**Table 2:** List of probable glaucoma-causing mutations identified from *MYOC* exon sequencing in 529 African American POAG subjects and 270 controls.

**Exon location**	**Amino acid change**	Nucleotide change*	**Number of cases**	**Number of controls**	**Age at diagnosis**	**Maximal IOP**	**Cup to disc ratio**
Exon 2	Thr209Asn	626G>T	1 (0.2%)	0	65	50	0.9
Exon 2	Leu215Gln	644A>T	2 (0.4%)	0	73, 72	23, 16	0.9, 0.9
Exon 3	Gln368X	1102G>A	1 (0.2%)	0	56	25	1.0
Exon 3	Thr377Met	1130G>A	1 (0.2%)	0	47	15	1.0
Exon 3	Ser393Arg	1179G>C	1 (0.2%)	0	75	31	1.0
Exon 3	Tyr453MetfsX11		1 (0.2%)	0	80	25	0.45

*Nucleotides numbered as in Ensembl accession number ENSG00000034971 (transcript ID ENST00000037502). IOP is for intraocular pressure. POAG is for primary open-angle glaucoma.

We also identified an additional twelve non-synonymous coding variants (Table 3), which appear to be neutral polymorphisms. Four of them (Arg76Lys, Val329Met, Glu352Lys, Lys398Arg) were previously reported as neutral polymorphisms [17]. All of the variants except for Val329Met were identified in both cases and controls. Val329Met was found in 2 cases but no control subjects. Two of the variants in exon 3, Thr353Ile and Lys500Arg, were previously reported with uncertain pathogenicity [17]. We identified Thr353Ile in only 1 control and the Lys500Arg variant in 4 cases and 2 controls. The remaining six variants were novel, including Ala108Gly, Arg126Gln, Arg226Gln, Gly244Ser, Ser333Cys, and Asp446Tyr [17]. Each of these novel variants was found in only 1 control subject, except for Ser333Cys, which was found in 1 case and 1 control.

**Table 3:** List of non-synonymous variants identified from *MYOC* exon sequencing in 529 African American POAG subjects and 270 controls.

**Exon location**	**Amino acid change**	Nucleotide change*	**dbSNP ID**	**Number of cases**	**Number of controls**	**p-value†**
Exon 1	Arg76Lys	227C>T	rs2234926	31 (5.9%)	14 (5.2%)	0.75
Exon 1	Ala108Gly	323G>C		0 (0.0%)	1 (0.4%)	0.34
Exon 1	Arg126Gln	377C>T		0 (0.0%)	1 (0.4%)	0.34
Exon 2	Arg226Gln	677C>T		0 (0.0%)	1 (0.4%)	0.34
Exon 2	Gly244Ser	730C>T		0 (0.0%)	1 (0.4%)	0.34
Exon 3	Val329Met	985C>T		2 (0.4%)	0 (0.0%)	0.55
Exon 3	Ser333Cys	997T>A		1 (0.2%)	1 (0.4%)	1.0
Exon 3	Glu352Lys	1054C>T	rs61745146	13 (2.5%)	5 (1.9%)	0.80
Exon 3	Thr353Ile	1058G>A		0 (0.0%)	1 (0.4%)	0.34
Exon 3	Lys398Arg	1193T>C	rs56314834	1 (0.2%)	1 (0.4%)	1.0
Exon 3	Asp446Tyr	1336C>A		0 (0.0%)	1 (0.4%)	0.34
Exon 3	Lys500Arg	1499T>C		4 (0.8%)	2 (0.7%)	1.0

*Nucleotides numbered as in Ensembl accession number ENSG00000034971 (transcript ID ENST00000037502). † Fisher’s exact test two-tailed p value.

In addition, we also identified 10 synonymous coding variants and one variant in the 5′-untranslated region (5′-UTR; Table 4). None of these silent variants were found to have a significant association with POAG. The 5′ UTR variant has not been previously reported and was only found in 1 control subject.

**Table 4:** List of synonymous and non-coding variants identified from *MYOC* exon sequencing in 529 African American POAG subjects and 270 controls.

**Location**	**Amino acid change**	Nucleotide change*	**dbSNP ID**	**Number of cases**	**Number of controls**	**p-value†**
Exon 1	Pro13Pro	39A>C	rs12082573	35 (6.6%)	8 (3.0%)	0.12
Exon 1	Gln101Gln	303T>C	rs61730978	1 (0.2%)	0 (0.0%)	1.0
Exon 1	Gly122Gly	366G>A		2 (0.4%)	0 (0.0%)	0.55
Exon 1	Leu159Leu	477T>C	rs61730977	70 (13.2%)	29 (10.7%)	0.36
Exon 2	Thr204Thr	612C>A	rs57824969	11 (2.1%)	3 (1.1%)	0.40
Exon 3	Thr285Thr	855C>A		0 (0.0%)	1 (0.4%)	0.34
Exon 3	Thr293Thr	879C>T		1 (0.2%)	0 (0.0%)	1.0
Exon 3	Thr325Thr	975C>T	rs61730976	61 (11.5%)	37 (13.7%)	0.42
Exon 3	Tyr347Tyr	1041A>G	rs61730974	6 (1.1%)	0 (0.0%)	0.10
Exon 3	Glu396Glu	1188C>T	rs61730975	37 (7.0%)	13 (4.8%)	0.28
5′ UTR		1515+4c>g		0 (0.0%)	1 (0.4%)	0.34

*Nucleotides numbered as in Ensembl accession number ENSG00000034971 (transcript ID ENST00000037502). † Fisher’s exact test two-tailed p value. SNP is for single nucleotide polymorphism.

Overall, the frequency of non-synonymous changes was not significantly different between cases and controls. Only one control subject was found to carry multiple non-synonymous variants of Asp446Tyr and Lys500Arg. The Asp446Tyr variant has not been previously reported and the Lys500Arg variant is considered to be of uncertain pathogenicity [17]. All other individuals had no more than 1 non-synonymous coding variant. Multiple synonymous variants did occur together frequently, more commonly in cases than in controls. These multiple synonymous variants were found in 10.8% of cases and 5.6% of controls, which was significantly different (Fisher’s exact test, p=0.02).

## Discussion

This study is the largest survey of myocilin mutations in African American subjects with and without POAG. We identified six myocilin mutations in seven POAG patients. The overall frequency of glaucoma-causing mutations in POAG subjects was 1.4%. Our finding is very similar with those in the Moroccan population [29], but is significantly lower than those in the Ghana and South African populations as well as in a prior study of an African American population [27,28,30]. Although our initial sequencing was done only in the forward direction, the high sequence quality and the complete coverage of the coding region minimized the possibility of missing any additional potential mutations. This should not contribute to the low prevalence of myocilin mutations in our study.

We identified two novel myocilin mutations located in exon 2. Up to date, only neutral polymorphisms and polymorphisms of uncertain pathogenicity have been reported in exon 2 [17]. The Thr209Asn mutation was found in 1 heterozygous POAG subject and in no controls. The Leu215Gln mutation was found in 2 heterozygous POAG subjects and in no controls. The 2 POAG subjects with the Leu215Gln variant were also heterozygous for either of the synonymous polymorphisms of Leu159Leu or Thr325Thr. According to the SIFT and Polyphen databases that predict effects of amino acid substitutions on MYOC structure and function [40,41], the Thr209Asn variant was determined to be benign while the Leu215Gln variant was determined to be probably damaging. More evidence is needed to determine whether the Thr209Asn mutation contributes to the development of glaucoma but current evidence suggests that the Leu215Gln variant is a novel glaucoma-causing mutation.

Two other non-synonymous variants that have not been previously reported are found only in controls. Each of the Asp446Tyr variant and the1515+4C>G variant in the 5′UTR are found in 1 heterozygous control. The difference in prevalence of these two variants between POAG subjects and controls is not statistically significant by the Fisher Exact Test (p>0.05). It is unclear why these two variants are overrepresented in controls but it is conceivable that they may have a protective role against the development of POAG. However, further studies are needed to replicate these findings. Although the frequency of several sequence variants differed between cases and controls, these differences were not statistically significant (Fisher’s exact test, p>0.05).

Comparing to the previous screen of myocilin mutations in African American POAG subjects and controls [30], our larger study not only corroborated their findings for three of the probable glaucoma-causing mutations (Gln368X, Ser393Arg, and Tyr453FS), but also identified one POAG subject with the Thr377Met mutation. This mutation is one of the most commonly found POAG-causing mutations and has been previously identified in populations from Australia, United States of America, Greece, the former Yugoslavian Republic of Macedonia, India, Finland, and Morocco [29,33,34,42-45]. Although the previous African American study found Glu352Lys variant in 2 POAG patients and no controls [30], we found this variant in 13 POAG patients and 5 controls that were heterozygous for the variant. The frequency in cases and controls was not statistically different by the Fisher’s Exact Test (p>0.05). Our data supported the reclassification of this variant as a polymorphism (dbSNP; rs61745146).

In summary, we have performed the largest screening of myocilin mutations in the African American population. The overall frequency of probable glaucoma-causing mutations in myocilin was lower in our data set of African American POAG subjects compared to prior reported frequencies of 2%–4% in many different populations [18]. This difference could be due to ascertainment bias due to recruitment of prevalent cases from glaucoma clinics compared with incident cases in a population-based study. Our study not only confirms the contribution of myocilin mutations in the African American population, but also suggests the greater genetic heterogeneity of POAG in this admixed population.

## References

[1] Resnikoff S, Pascolini D, Etya'ale D, Kocur I, Pararajasegaram R, Pokharel GP, Mariotti SP (2004). Global data on visual impairment in the year 2002.. Bull World Health Organ.

[2] Congdon N, O'Colmain B, Klaver CC, Klein R, Munoz B, Friedman DS, Kempen J, Taylor HR, Mitchell P (2004). Causes and prevalence of visual impairment among adults in the United States.. Arch Ophthalmol.

[3] Quigley HA, Broman AT (2006). The number of people with glaucoma worldwide in 2010 and 2020.. Br J Ophthalmol.

[4] Liu Y, Allingham RR (2011). Molecular genetics in glaucoma.. Exp Eye Res.

[5] Allingham RR, Liu Y, Rhee DJ (2009). The genetics of primary open-angle glaucoma: a review.. Exp Eye Res.

[6] Tielsch JM, Sommer A, Katz J, Royall RM, Quigley HA, Javitt J (1991). Racial variations in the prevalence of primary open-angle glaucoma. The Baltimore Eye Survey.. JAMA.

[7] Tielsch JM, Katz J, Sommer A, Quigley HA, Javitt JC (1994). Family history and risk of primary open angle glaucoma. The Baltimore Eye Survey.. Arch Ophthalmol.

[8] Fingert JH (2011). Primary open-angle glaucoma genes.. Eye (Lond).

[9] Liu Y, Allingham RR. Genetics of Glaucoma. Encyclopeida of Life Sciences (ELS). Chichelster: John Wiley & Sons, Ltd.; 2010.

[10] Sheffield VC, Stone EM, Alward WL, Drack AV, Johnson AT, Streb LM, Nichols BE (1993). Genetic linkage of familial open angle glaucoma to chromosome 1q21-q31.. Nat Genet.

[11] Stone EM, Fingert JH, Alward WL, Nguyen TD, Polansky JR, Sunden SL, Nishimura D, Clark AF, Nystuen A, Nichols BE, Mackey DA, Ritch R, Kalenak JW, Craven ER, Sheffield VC (1997). Identification of a gene that causes primary open angle glaucoma.. Science.

[12] Rezaie T, Child A, Hitchings R, Brice G, Miller L, Coca-Prados M, Heon E, Krupin T, Ritch R, Kreutzer D, Crick RP, Sarfarazi M (2002). Adult-onset primary open-angle glaucoma caused by mutations in optineurin.. Science.

[13] Monemi S, Spaeth G, DaSilva A, Popinchalk S, Ilitchev E, Liebmann J, Ritch R, Heon E, Crick RP, Child A, Sarfarazi M (2005). Identification of a novel adult-onset primary open-angle glaucoma (POAG) gene on 5q22.1.. Hum Mol Genet.

[14] Stoilov I, Akarsu AN, Sarfarazi M (1997). Identification of three different truncating mutations in cytochrome P4501B1 (CYP1B1) as the principal cause of primary congenital glaucoma (Buphthalmos) in families linked to the GLC3A locus on chromosome 2p21.. Hum Mol Genet.

[15] Kawase K, Allingham RR, Meguro A, Mizuki N, Roos B, Solivan-Timpe FM, Robin AL, Ritch R, Fingert JH (2012). Confirmation of TBK1 duplication in normal tension glaucoma.. Exp Eye Res.

[16] Fingert JH, Robin AL, Stone JL, Roos BR, Davis LK, Scheetz TE, Bennett SR, Wassink TH, Kwon YH, Alward WL, Mullins RF, Sheffield VC, Stone EM (2011). Copy number variations on chromosome 12q14 in patients with normal tension glaucoma.. Hum Mol Genet.

[17] Hewitt AW, Mackey DA, Craig JE (2008). Myocilin allele-specific glaucoma phenotype database.. Hum Mutat.

[18] Fingert JH, Stone EM, Sheffield VC, Alward WL (2002). Myocilin glaucoma.. Surv Ophthalmol.

[19] Fingert JH, Ying L, Swiderski RE, Nystuen AM, Arbour NC, Alward WL, Sheffield VC, Stone EM (1998). Characterization and comparison of the human and mouse GLC1A glaucoma genes.. Genome Res.

[20] Kwon YH, Fingert JH, Kuehn MH, Alward WL (2009). Primary open-angle glaucoma.. N Engl J Med.

[21] Kubota R, Noda S, Wang Y, Minoshima S, Asakawa S, Kudoh J, Mashima Y, Oguchi Y, Shimizu N (1997). A novel myosin-like protein (myocilin) expressed in the connecting cilium of the photoreceptor: molecular cloning, tissue expression, and chromosomal mapping.. Genomics.

[22] Jacobson N, Andrews M, Shepard AR, Nishimura D, Searby C, Fingert JH, Hageman G, Mullins R, Davidson BL, Kwon YH, Alward WL, Stone EM, Clark AF, Sheffield VC (2001). Non-secretion of mutant proteins of the glaucoma gene myocilin in cultured trabecular meshwork cells and in aqueous humor.. Hum Mol Genet.

[23] Zhou Z, Vollrath D (1999). A cellular assay distinguishes normal and mutant TIGR/myocilin protein.. Hum Mol Genet.

[24] Resch ZT, Fautsch MP (2009). Glaucoma-associated myocilin: a better understanding but much more to learn.. Exp Eye Res.

[25] Gould DB, Reedy M, Wilson LA, Smith RS, Johnson RL, John SW (2006). Mutant myocilin nonsecretion in vivo is not sufficient to cause glaucoma.. Mol Cell Biol.

[26] Gong G, Kosoko-Lasaki O, Haynatzki GR, Wilson MR (2004). Genetic dissection of myocilin glaucoma.. Hum Mol Genet.

[27] Whigham BT, Williams SE, Liu Y, Rautenbach RM, Carmichael TR, Wheeler J, Ziskind A, Qin X, Schmidt S, Ramsay M, Hauser MA, Allingham RR (2011). Myocilin mutations in black South Africans with POAG.. Mol Vis.

[28] Challa P, Herndon LW, Hauser MA, Broomer BW, Pericak-Vance MA, Ababio-Danso B, Allingham RR (2002). Prevalence of myocilin mutations in adults with primary open-angle glaucoma in Ghana, West Africa.. J Glaucoma.

[29] Melki R, Idhajji A, Driouiche S, Hassani M, Boukabboucha A, Akhayat O, Garchon H, Belmouden A (2003). Mutational analysis of the Myocilin gene in patients with primary open-angle glaucoma in Morocco.. Ophthalmic Genet.

[30] Fingert JH, Heon E, Liebmann JM, Yamamoto T, Craig JE, Rait J, Kawase K, Hoh ST, Buys YM, Dickinson J, Hockey RR, Williams-Lyn D, Trope G, Kitazawa Y, Ritch R, Mackey DA, Alward WL, Sheffield VC, Stone EM (1999). Analysis of myocilin mutations in 1703 glaucoma patients from five different populations.. Hum Mol Genet.

[31] Ennis S, Gibson J, Griffiths H, Bunyan D, Cree AJ, Robinson D, Self J, MacLeod A, Lotery A (2010). Prevalence of myocilin gene mutations in a novel UK cohort of POAG patients.. Eye (Lond).

[32] Hulsman CA, De Jong PT, Lettink M, Van Duijn CM, Hofman A, Bergen AA (2002). Myocilin mutations in a population-based sample of cases with open-angle glaucoma: the Rotterdam Study.. Albrecht Von Graefes Arch Klin Exp Ophthalmol.

[33] Kanagavalli J, Krishnadas SR, Pandaranayaka E, Krishnaswamy S, Sundaresan P (2003). Evaluation and understanding of myocilin mutations in Indian primary open angle glaucoma patients.. Mol Vis.

[34] Wiggs JL, Allingham RR, Vollrath D, Jones KH, De La Paz M, Kern J, Patterson K, Babb VL, Del Bono EA, Broomer BW, Pericak-Vance MA, Haines JL (1998). Prevalence of mutations in TIGR/Myocilin in patients with adult and juvenile primary open-angle glaucoma.. Am J Hum Genet.

[35] Faucher M, Anctil JL, Rodrigue MA, Duchesne A, Bergeron D, Blondeau P, Cote G, Dubois S, Bergeron J, Arseneault R, Morissette J, Raymond V (2002). Founder TIGR/myocilin mutations for glaucoma in the Quebec population.. Hum Mol Genet.

[36] Miller SA, Dykes DD, Polesky HF (1988). A simple salting out procedure for extracting DNA from human nucleated cells.. Nucleic Acids Res.

[37] Liu Y, Schmidt S, Qin X, Gibson J, Hutchins K, Santiago-Turla C, Wiggs JL, Budenz DL, Akafo S, Challa P, Herndon LW, Hauser MA, Allingham RR (2008). Lack of association between LOXL1 variants and primary open-angle glaucoma in three different populations.. Invest Ophthalmol Vis Sci.

[38] Liu Y, Akafo S, Santiago-Turla C, Cohen CS, Larocque-Abramson KR, Qin X, Herndon LW, Challa P, Schmidt S, Hauser MA, Allingham RR (2008). Optineurin coding variants in Ghanaian patients with primary open-angle glaucoma.. Mol Vis.

[39] Rozen S, Skaletsky H (2000). Primer3 on the WWW for general users and for biologist programmers.. Methods Mol Biol.

[40] Ng PC, Henikoff S (2003). SIFT: Predicting amino acid changes that affect protein function.. Nucleic Acids Res.

[41] Ramensky V, Bork P, Sunyaev S (2002). Human non-synonymous SNPs: server and survey.. Nucleic Acids Res.

[42] Mackey DA, Healey DL, Fingert JH, Coote MA, Wong TL, Wilkinson CH, McCartney PJ, Rait JL, de Graaf AP, Stone EM, Craig JE (2003). Glaucoma phenotype in pedigrees with the myocilin Thr377Met mutation.. Arch Ophthalmol.

[43] Shimizu S, Lichter PR, Johnson AT, Zhou Z, Higashi M, Gottfredsdottir M, Othman M, Moroi SE, Rozsa FW, Schertzer RM, Clarke MS, Schwartz AL, Downs CA, Vollrath D, Richards JE (2000). Age-dependent prevalence of mutations at the GLC1A locus in primary open-angle glaucoma.. Am J Ophthalmol.

[44] Puska P, Lemmela S, Kristo P, Sankila EM, Jarvela I (2005). Penetrance and phenotype of the Thr377Met Myocilin mutation in a large Finnish family with juvenile- and adult-onset primary open-angle glaucoma.. Ophthalmic Genet.

[45] Petersen MB, Kitsos G, Samples JR, Gaudette ND, Economou-Petersen E, Sykes R, Rust K, Grigoriadou M, Aperis G, Choi D, Psilas K, Craig JE, Kramer PL, Mackey DA, Wirtz MK (2006). A large GLC1C Greek family with a myocilin T377M mutation: inheritance and phenotypic variability.. Invest Ophthalmol Vis Sci.

